# Microflow Liquid Chromatography Coupled to Multinozzle Electrospray Ionization for Improved Lipidomics Coverage of 3D Clear Cell Renal Cell Carcinoma

**DOI:** 10.1021/acs.analchem.4c06337

**Published:** 2025-02-25

**Authors:** Sergey Girel, Mathieu Galmiche, Mathis Fiault, Valentin Mieville, Patrycja Nowak-Sliwinska, Serge Rudaz, Isabel Meister

**Affiliations:** 1 School of Pharmaceutical Sciences, University of Geneva, Geneva 4 1211, Switzerland; 2 Institute of Pharmaceutical Sciences of Western Switzerland, University of Geneva, Geneva 4 1211, Switzerland; 3 Swiss Center of Applied Human Toxicology (SCAHT), Basel 4000, Switzerland

## Abstract

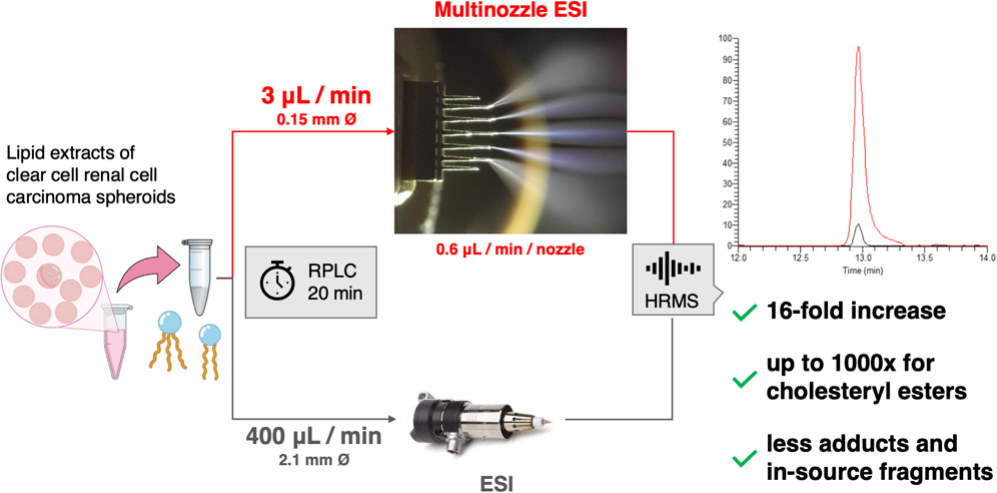

In most bioanalytical laboratories, high-resolution mass spectrometry (HRMS) systems with electrospray ionization (ESI) are hyphenated to liquid chromatography platforms. The latter typically operate under analytical flow (AF; 0.2–1 mL/min) regimes. Hence, AF/ESI-HRMS methods prioritize the detection of analytes of higher abundances or ionizability and tend to suffer from matrix effects or ion suppression. A far higher sensitivity can be obtained with electrospray at nanoflow (10–1000 nL/min) thanks to a better ionization efficiency and significant decrease in matrix effects. Both advantages are crucial to reliably accessing low-abundance compounds or weakly ionizable analytes. This work presents a microflow (μF) chromatographic setup coupled to a novel microfabricated multinozzle electrospray (mnESI) emitter with five nozzles spraying at 600 nL/min per nozzle for untargeted HRMS lipidomic profiling. With a runtime of 19 min, similar to our established analytical flow (AF/ESI) lipidomics platform, μF/mnESI produced a 16-fold median increase across 69 deuterated lipid standards. The performance of this new configuration was also evaluated in the context of the profiling of a 3D clear cell renal cell carcinoma (ccRCC) model exposed to a multidrug combination therapy. The processing of the acquired data resulted in 1270 (μF/mnESI) vs 752 (AF/ESI) MS^2^-annotated lipids. Among those, 762 achieved <10% variation on pooled QC samples for μF/mnESI compared to only 361 for the AF method. In addition, the measurements of ccRCC samples confirmed the improvements in ionization efficiency and adduct patterns observed with standards, enabling to annotate 79 oxidized triglycerides, 38 cholesterol esters (only five and four detected in AF/ESI, respectively), and 12 sitosterol esters, not yet reported in mammalian cell cultures.

## Introduction

Lipid systems biology, i.e., the identification of lipid molecular profiles, their dynamics, and associated biological pathways, requires the acquisition of complex data sets from different experimental sources. Lipidomic analysis has become a rapidly growing field among -omics sciences and a major challenge for analytical chemistry per se, owing to the structural diversity of the lipidome. Due to a wide dynamic range of concentrations spanning from pM to μM,^1^ the analysis is predominantly conducted with electrospray ionization mass spectrometry (ESI-MS) methods.^2^ Although discovery approaches put an emphasis on lipid alteration patterns in a systemic context, description and thorough structural characterization of the highest possible number of molecular species remain an important task. Therefore, liquid chromatography (LC) is coupled with MS detection to facilitate the separation of isomers and decrease the strong ion suppression originating from the multitude of coeluting compounds.^3,4^

To date, most LC/ESI-MS experiments in discovery lipidomics used analytical-bore column dimensions with a 2.1 mm internal diameter (ID) and up to 150 mm length at 200–500 μL/min flow rates. Reversed-phase (RP) separation using fully or superficially porous silica functionalized with hydrophobic ligands (C_4_ to C_30_) remains a method of choice due to the well-studied separation mechanisms and overall robustness of the technique.^3,5^ Recently, several groups demonstrated that nanoscale LC coupling with nanoelectrospray ionization (nLC/NSI-MS), widely utilized in protein biomarker research, may also be a valuable analytical configuration for lipidomic studies. It is especially suitable for deep screening of biological lipid extracts due to benefits of nLC/NSI-MS, such as reduced chromatographic dilution, improved ionization efficiency, low sample and solvent consumption, and broader coverage of the lipidome.^6−9^ However, nLC suffers from practical operational problems in the form of hardly detectable leaks, large influence of system volumes, and associated longer runtimes. In addition, well-trained and skilled scientists are needed to operate this costly nanoflow equipment. Therefore, the bioanalytical community is, in general, still scared away from implementing nLC-based methods in routine workflows.

Microflow LC (μF) using flow rates of 1–50 μL/min appears as an excellent strategy to reduce the detrimental effects of system volumes and increase sample loading and overall system robustness while achieving throughput levels comparable to conventional analytical-bore methods.^40^ As a drawback, the associated ionization efficiency and ion sampling improvements are significantly lower in μF compared to nLC/NSI, making it harder to justify the transition.^10^ Thanks to new microfabricated monolithic multinozzle (M3) electrospray emitters, it becomes possible to effectively obtain NSI from μF separation via at-source splitting of the incoming effluent.^11^ In this work, we evaluate how this μF/mnESI setup enabled by M3 emitters could be considered for discovery lipidomics. Its capacity to achieve deep lipidome profiling of biological samples in a time frame comparable to classic LC/ESI-MS measurements is next demonstrated in a proof-of-concept application involving a comparative analysis of lipid extracts from 3D clear cell renal cell carcinoma (ccRCC). ccRCC is among the most difficult-to-treat cancers due to high occurrences of treatment resistance and adverse effects of chemotherapy. We investigated the lipid profiles of ccRCC cells in three-dimensional culture conditions (3D) exposed to an optimized proprietary multidrug combination therapy using treatment-naive and treatment-resistant phenotypes.^12,13^

## Materials and Methods

### Chemicals

Water, acetonitrile, and isopropanol (Optima LC/MS) were purchased from Thermo Fisher Scientific (Waltham, Massachusetts, USA). Ammonium acetate (LiChropur) and DMSO were provided by Merck (Darmstadt, Germany). LC/MS-grade acetic acid was obtained from Biosolve (Dieuze, France). The mix of 69 deuterated lipid standards was purchased from Avanti Polar Lipids (UltimateSPLASH ONE, Alabaster, Alabama, USA). Mass spectrometer calibration solution was supplied by Thermo Fisher Scientific.

### Sample Preparation

Cell samples were prepared, treated, and harvested as described in the supplementary information (Document S1). The sample processing was conducted in two steps, as described in the supplementary information (Document S1). Briefly, after a polar metabolite extraction using methanol:water solution (4:1, *v*/*v*), lipids were extracted from the remaining pellet with 100% isopropanol. Shortly before analysis, the dried lipid samples were reconstituted in 120 μL of methanol and shaken for 15 min at 1200 rpm/4 °C. Prior to the transfer to Waters Total Recovery glass vials (Milford, Massachusetts, USA), the samples were centrifuged for 15 min at 14,000*g* and 4 °C. The pooled quality control sample was created by aliquoting 20 μL of each sample with subsequent thorough mixing. For the evaluation of μF/mnESI ionization, dynamic range, and adduction patterns, the Ultimate SPLASH ONE mix of 69 deuterated lipids was diluted 50-, 500-, 5000-, 50,000- and 500,000-fold with methanol. For the resulting concentrations, see Table S1.

### Microflow/mnESI Method

Chromatographic separation was performed on a Bruker PepSep column (0.15 × 150 mm, ReproSil-Pur C_18_-AQ 1.9 μm, 120 Å, C-load 15%; Billerica, Massachusetts, USA) in a Waters M-Class system composed of a μBSM binary pump with an embedded flow controller, a μSM-FL fixed loop sampler, and a TVM module equipped with switching valves and a heated column compartment. The injection volume was set to 0.2 μL using the partial loop direct injection mode. The calculated delay volume from the flow controller outlet to the column inlet was 1.76 μL with all the fluidic connections created using Security Link fittings (25 μm, Phenomenex, Torrance, California, USA). Mobile phases were (A) acetonitrile:water + 0.1% acetic acid (1:1 v/v, 10 mM ammonium acetate) and (B) isopropanol:acetonitrile + 0.1% acetic acid (8:2 v/v, 10 mM ammonium acetate). The gradient program ran at 3 μL/min from 3 to 99% B in 11.5 min for a total run time of 19 min. The detection was carried out using an online hyphenated Orbitrap Exploris 120 mass spectrometer (Thermo Fisher Scientific) equipped with a DuoESI source containing a five-nozzle (20 μm nozzle) or an eight-nozzle (10 μm nozzle) M3 silicon emitter (Newomics, Berkeley, California, USA). Connection of the column outlet to the emitter was performed using a 35 cm nanoViper capillary (20 μm, Thermo Fisher Scientific) according to the manufacturer’s instructions. The analysis was performed in positive polarity (4.5 kV for the five-nozzle emitter and 3.5 kV for the eight-nozzle emitter) in DDA mode over a 400–1200 Da scan range and resolution set to 60,000 for MS^1^ and 15,000 for MS^2^ at *m*/*z* 200. Sheath gas was set at 6 au, while auxiliary and sweep gases were turned off. The transfer tube temperature was set at 300 °C, the RF lens at 70%, the ITT at 80 ms, and the AGC at 200%. The stage position was set at 0 mm on the *X*-axis, +2.7 mm or +3.5 on the *Y*-axis for the five- or the eight-nozzle emitter, respectively, and the *Z*-axis slightly adjusted to put the emitter in the same plane as the MS inlet. The targeted mass exclusion list for MS/MS acquisition was based on lists provided by Keller et al.^14^ and Rardin.^15^ The full description of the method parameters is provided in the Supporting Information (Table S2 for the μF/mnESI and Table S3 for the AF/ESI).

The analytical sequence used for batch-proofing the μF/mnESI method was constituted of 42 biological samples, 8 pooled QCs, and 8 twofold diluted QCs, with a system conditioning consisting of 20 injections of QC material. To investigate the influence of injecting diluted material on the following sample, each diluted QC was followed by a reconditioning pooled QC (μF/mnESI and AF/ESI sequences are detailed in Table S4).

### Data Preprocessing

For the evaluation of in-source fragmentation (ISF), MS/MS spectra of the standards obtained from intact M+H^+^ or M+NH_4_^+^ precursors were manually extracted in Xcalibur Qual Browser (ver. 4.0.27.13, Thermo Fisher Scientific). As they were obtained under rather mild conditions (NCE 20%), the most abundant fragment peak was considered as a candidate to assess ISF. Next, the averaged accurate mass of the fragment across collected MS^2^ spectra was extracted from the full-scan TIC trace under the 5 ppm mass tolerance value and the resulting chromatographic peak was considered if coeluting with the selected ion chromatogram of the parent ion. Finally, fragment-to-precursor ion peak area ratios were used as a measure of ISF.

For all other measurements, the raw data (Thermo.RAW format) were deconvoluted with the open-source MS-DIAL package^16^ (v4.92) using the parameters described in Table S5. Lipid annotation was conducted on the species level as outlined by recent LIPID MAPS classification, and the shorthand notation update by Liebisch et al.^17^ Molecular species-level assignments were provided where available. The annotated data were manually checked to correct automatic integration and remove unreliable hits demonstrating either unsatisfactory chromatographic performance (massively distorted, smeared, split peaks) or noninformative fragmentation spectra. Intensity drift along the analytical sequence was corrected using LOESS regression^18,19^ for each feature via pooled QC data (tricube kernel, initial span 0.75). Peak areas were taken as a measure of concentration, and areas of all adducts were summed for each annotated lipid to provide final abundance values.^20,21^

### Data Analysis

Multivariate analysis of the 3D cell sequence was conducted by using SIMCA 16 (Umetrics Sartorius Stedim, Umeå, Sweden). The data matrices were mean-centered and scaled to unit variance for data exploration using an embedded principal component analysis module (PCA-X) and for data analysis with supervised models. PCA-X score plots and loadings were used to compare analytical quality and overall data structure of both setups. Due to the high sample-to-sample variability of the spheroids relative to biological responses, lipidomic profiles were investigated using orthogonal partial least squares discriminant analysis (OPLS-DA).^22^ Lipids with CV_QC_ < 10% were used to build OPLS-DA models. Shifts associated with exposure were compared between naive and resistant cells using shared-and-unique structure (SUS) plots. SUS plots are graphical tools combining correlation loading from the predictive components of two OPLS-DA models in the *x*- and *y*-axes and are particularly useful to highlight differences that are common or specific between two conditions compared to controls.

## Results

### Setup Configuration

Fused silica capillaries with IDs of 150–200 μm were considered as optimal columns for the current experiments due to their operation range of 2 to 5 μL/min flow rates. With a five- or eight-nozzle emitter, the resulting flow per nozzle was reduced to the sub-μL range, enabling a multiplexed nanoelectrospray. Next, the 150 mm length was chosen to minimize the ratio of the injection-to-column volumes for good chromatographic performance while achieving tolerable system backpressure. To our knowledge, the only commercially available column with these specifications was the Bruker PepSep C_18_-AQ 0.15 × 150 mm with a pressure tolerance up to 1000 bar. We calculated the flow rate for this stationary phase to be 3 μL/min to achieve the targeted method runtime. The recommended nozzle design was the eight-nozzle emitter (10 μm ID), resulting in 0.375 μL/min/nozzle. To reduce the probability of clogging over a sequence with the biological matrix, the five-nozzle design (20 μm ID) providing 0.6 μL/min/nozzle was also tested. The mobile phase composition was adapted from the commonly used 60% acetonitrile in water (A) and 90% isopropanol in acetonitrile (B) to 50% acetonitrile (A) and 80% isopropanol (B), respectively, to decrease viscosity and to limit the maximal working system pressure to below 800 bar (tubing contribution of ∼200 bar). The gradient started with an isocratic step of 1 min at 3% B to allow sufficient time to separate early eluting lipids from the initial flow-through eluate, which displays increased nanospray instability. The gradient program was adapted to obtain an elution pattern of major lipid classes and a run time similar to our in-house conventional lipidomics method (i.e., 19 min). The optimization of the M3 emitter placement was achieved together with careful voltage ramping at initial gradient conditions for maximizing spray stability and ion sampling while avoiding discharges. Total current was kept below 10 μA (4.5–6 μA observed across the gradient). Sheath gas flow was adjusted to avoid the accumulation of droplets at the nozzle tips. Finally, the lower mass limit of the survey scans was raised from *m*/*z* 220 to 400 to avoid the environmental contaminant ions of *m*/*z* 327.008 and 371.316, which affect the available C-trap capacity.

### Evaluation of Ionization Behavior and Adduction Patterns in Standard Solutions

**Figure 1:**
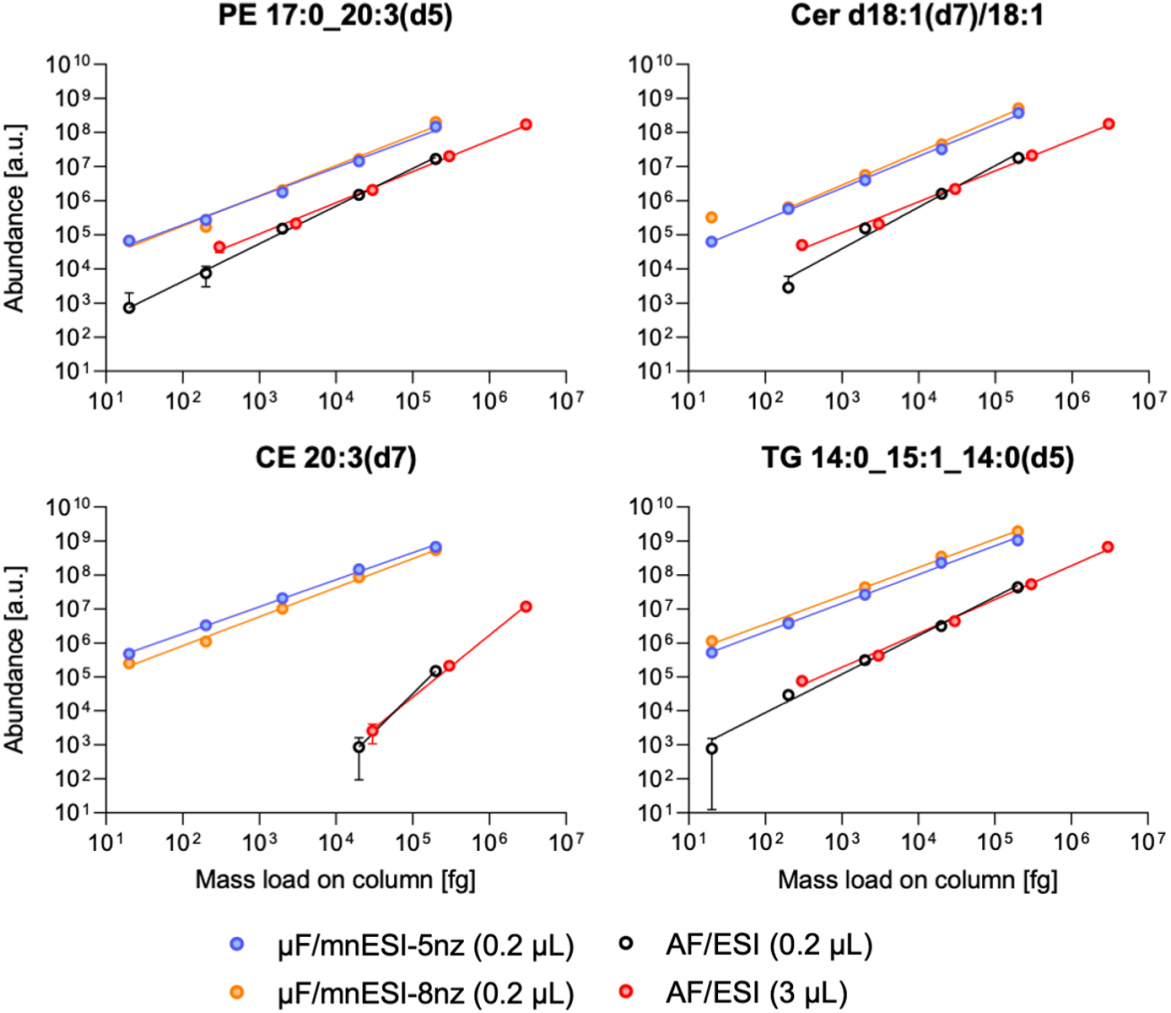
Dilution series data of selected lipids from the UltimateSPLASH ONE mix (plotted with GraphPad Prism v.9.5.1; mean, SD, log10 scale).

A dilution series of the UltimateSPLASH ONE mix of 69 deuterated lipid standards was used as a benchmark to investigate the ionization and adduction behaviors of the new setup. The μF/mnESI in both the five- and eight-nozzle configurations was compared to the AF/ESI platform first using the same injection volume (i.e., 0.2 μL). A 16-fold median increase in ion abundances in the five-nozzle configuration was observed compared to AF/ESI, with a particular gain for CEs of up to three orders of magnitude (Figure 1, Figure S1, and Table S3). The eight-nozzle configuration presented a 24-fold median increase without a further notable rise for the CE class. As AF workflows work with higher mass loads compared to capillary setups, the effective gain was estimated by comparing the μF/mnESI setup (0.2 μL injection) with our AF/ESI protocol based on a 3 μL volume injection. We obtained a 1.2-fold effective improvement across all of the standards. Notably, CEs displayed a gain of one to three orders of magnitude. Linearity across the dilution range was comparable between the methods. The ISF behavior of the mnESI exhibited significant differences compared to the high-flow ESI by offering much milder conditions for lipid classes such as PI, PGs, and Cer (Table 1).

**Table 1:** Comparison of the in-Source Fragmentation Based on One Main Fragment for Each Selected Representative Lipid between μF/mnESI (0.2 μL Injection) and AF/ESI (3 μL Injection) from Triplicate Injections

			fragment/precursor ratio (%, *n* = 3)		
lipid standard (adduct)	concentration (μg/mL)	fragment, measured *m*/*z*	μF/mnESI (five nozzles)	μF/mnESI (eight nozzles)	AF/ESI
PE 17:0_14:1(d5) (M+H^+^)	0.5	540.5022	2.8 ± 0.2	4.2 ± 0.3	4.2 ± 0.1
PE 17:0_20:3(d5) (M+H^+^)	1.0	568.5334	2.8 ± 0.1	4.0 ± 0.5	3.5 ± 0.1
PI 17:0_16:1(d5) (M+H^+^)	1.0	568.5341	11.7 ± 0.3	12.9 ± 0.1	294.1 ± 1.9
PG 17:0_20:3(d5) (M+NH_4_^+^)	1.0	620.5655	12.0 ± 0.3	13.7 ± 0.3	149.1 ± 1.3
Cer d18:1(d7)/20:1 (M+H^+^)	0.5	581.5990	26.2 ± 0.6	27.5 ± 0.7	111.7 ± 0.5
DG 17:0_18:1(d5) (M+NH_4_^+^)	1.5	596.5652	14.8 ± 0.1	14.9 ± 0.1	18.9 ± 0.6
TG 14:0_15:1_14:0(d5) (M+NH_4_^+^)	1.0	512.4710	2.1 ± 0.1	2.2 ± 0.1	3.3 ± 0.1

The adduction patterns of the injected standards also showed marked differences between the two flow regimens (Table 2). In the AF/ESI method, the protonated/ammoniated ion abundances were moderately higher than the sodium adducts or water losses (up to 10× higher peak areas). PGs, Cer, DGs, and CEs displayed nearly similar intensities of the main ion compared to their sodiated or water-loss counterparts. In contrast, the μF/mnESI method clearly favored the protonated/ammoniated species. This phenomenon was particularly striking for TGs and CEs, whose abundances as ammoniated adducts were increased 20–80× compared to sodium species. The eight-nozzle emitter, which provided almost half of the flow rate at nebulization (375 vs 600 nL/min), produced a further decrease in sodium adduct signals.

**Table 2:** Response Ratios (CV, %) of the Main Ion to the Adduct or Fragment Species in the μF/mnESI (0.2 μL Injection) Compared to the AF/ESI Method (3 μL Injection) from Triplicate Injections

			main ion/adduct ratio (*n* = 3)		
adduct	lipid standard species (main ion)	concentration (μg/mL)	μF/mnESI (five nozzles)	μF/mnESI (eight nozzles)	AF/ESI
sodium	LPE 17:0(d5) (M+H^+^)	1.0	9.7 (1.8)	22.1 (8.4)	8.2 (0.5)
	LPG 17:0(d5) (M+H^+^)	1.0	3.1 (3.5)	5.3 (10.1)	1.7 (1.8)
	PE 17:0_20:3(d5) (M+H^+^)	1.0	6.7 (10.1)	14.0 (2.3)	8.9 (0.9)
	PG 17:0_20:3(d5) (M+NH_4_^+^)	1.0	4.0 (4.3)	11.1 (8.2)	2.6 (0.7)
	SM d18:1/16:1(d9) (M+H^+^)	1.5	8.4 (2.7)	20.7 (6.3)	6.7 (1.4)
	Cer d18:1(d7)/20:1 (M+H^+^)	0.5	3.3 (4.0)	5.2 (10.1)	2.6 (1.1)
	DG 17:0_18:1(d5) (M+NH_4_^+^)	1.5	6.2 (6.7)	13.7 (4.1)	0.8 (1.7)
	TG 14:0_15:1_14:0(d5) (M+NH_4_^+^)	1.0	28.7 (4.8)	58.7 (1.8)	9.6 (1.3)
	TG 16:0_15:1_16:0(d5) (M+NH_4_^+^)	2.0	54.8 (1.0)	112.1 (3.3)	10.7 (0.5)
	CE 16:1(d7) (M+NH_4_^+^)	1.0	71.8 (3.7)	146.0 (6.7)	1.1 (5.1)
	CE 20:3(d7) (M+NH_4_^+^)	1.0	79.9 (7.4)	179.3 (3.3)	2.0 (5.1)
water loss	LPG 17:0(d5) (M+H^+^)	1.0	2.7 (0.3)	2.5 (1.1)	0.8 (1.3)
	Cer d18:1(d7)/20:1 (M+H^+^)	0.5	3.9 (3.0)	3.7 (2.9)	1.2 (0.2)

### Coverage of the ccRCC Cellular Lipidome

**Figure 2:**
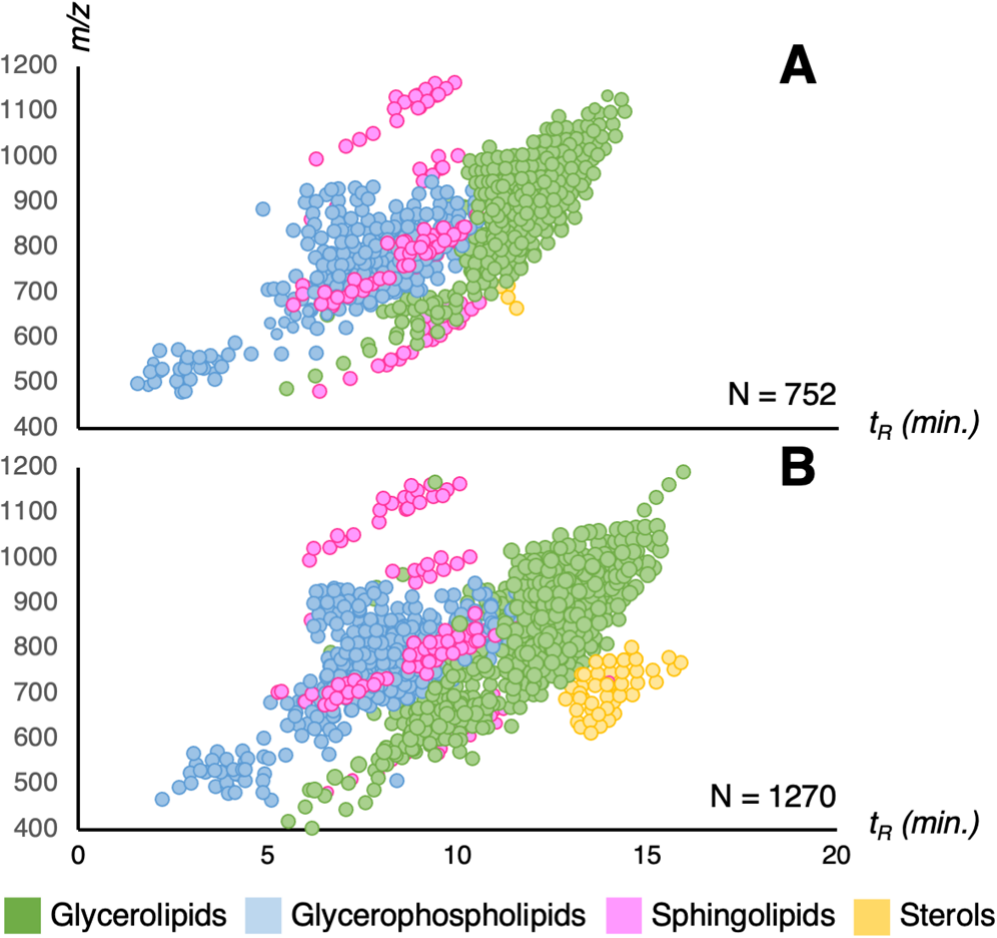
Overview of MS^2^-annotated lipids after data preprocessing with MS-DIAL and manual curation. (A) AF/ESI 2.1 × 100 mm BEH C18 column (400 μL/min, 3 μL injection). (B) μF/mnESI 0.15 × 150 mm PepSep C18-Aq column (3 μL/min, 0.2 μL injection).

The novel μF/mnESI approach produced 1270 annotated lipids, in contrast to the 752 annotations obtained from the AF/ESI lipidomics data. The μF/mnESI lipid maps (*t*_R_*vsm*/*z*) featured a notably higher number of sterols (38 CEs vs 4 in AF/ESI), which formed an easily recognizable cluster (Figure 2). Four additional lipid subclasses were only detected and unambiguously annotated by μF/mnESI: monoacylglycerols (MG, *n* = 9), monogalactosyldiacylglycerols (MGDG and ether MGDG, *n* = 3), digalactosyldiacylglycerols (DGDG, *n* = 3), and sitosterol esters (SISE, *n* = 12). In addition, a significantly higher number of oxidized triacylglycerols (oxTG) were detected (79 vs 5 in AF/ESI). Comprehensive data can be consulted in Tables S7 and S8.

**Figure 3:**
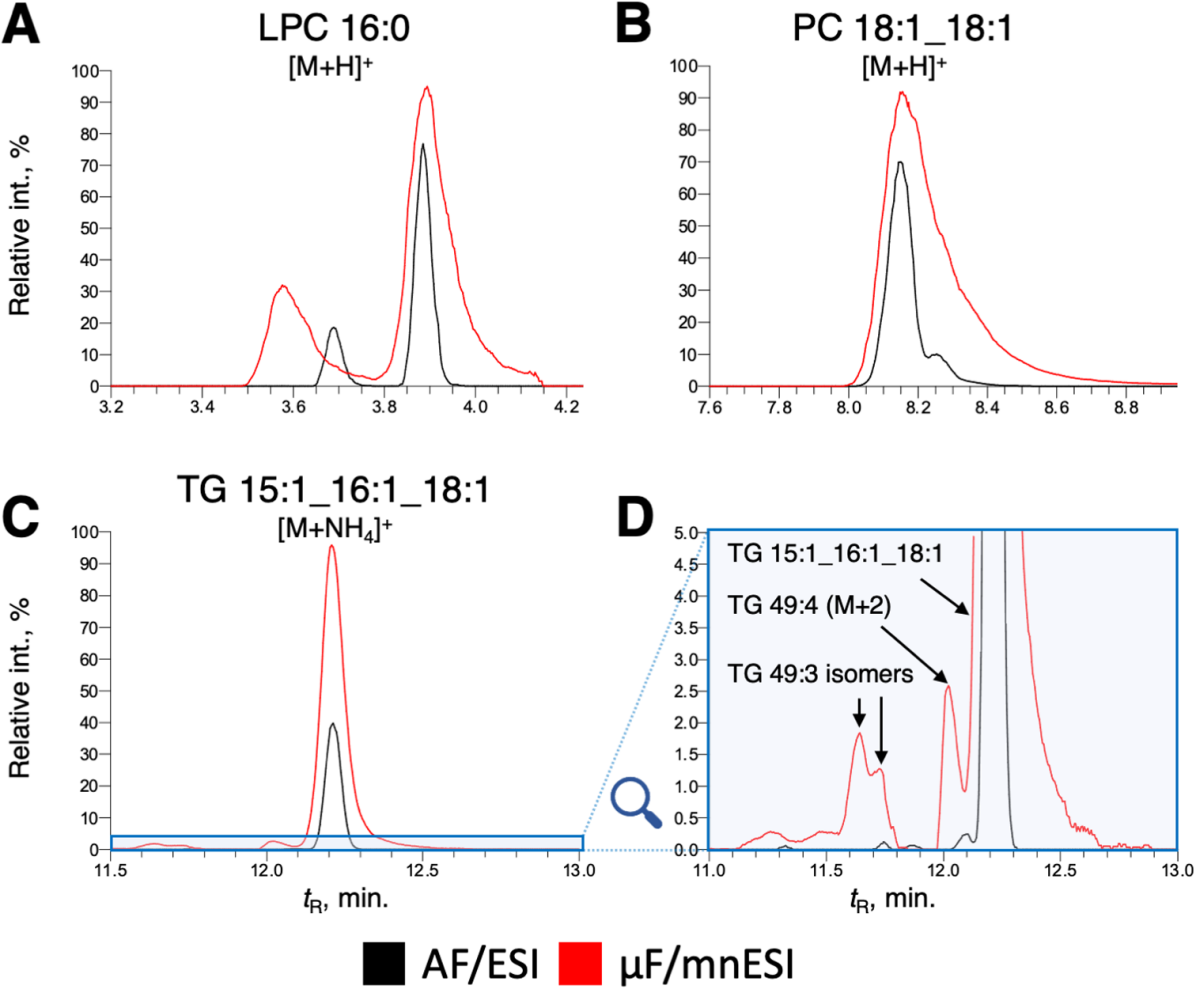
Comparative analysis of selected chromatographic peaks between the AF/ESI approach (black trace) and the μF/mnESI setup (red trace). (A) Separation of early eluting LysoPC 16:0 (*m*/*z* 496.339). (B) Loss of chromatographic resolution for isomers of PC 36:2 (*m*/*z* 786.600). (C) Separation of TG 49:3 (*m*/*z* 880.716). (D) Improved electrospray efficiency in the μF/mnESI method reveals low-abundance isomers of TG 49:3 and an interference from the M+2 isotope peak of TG 49:4, which was filtered out at the preprocessing stage.

**Figure 4:**
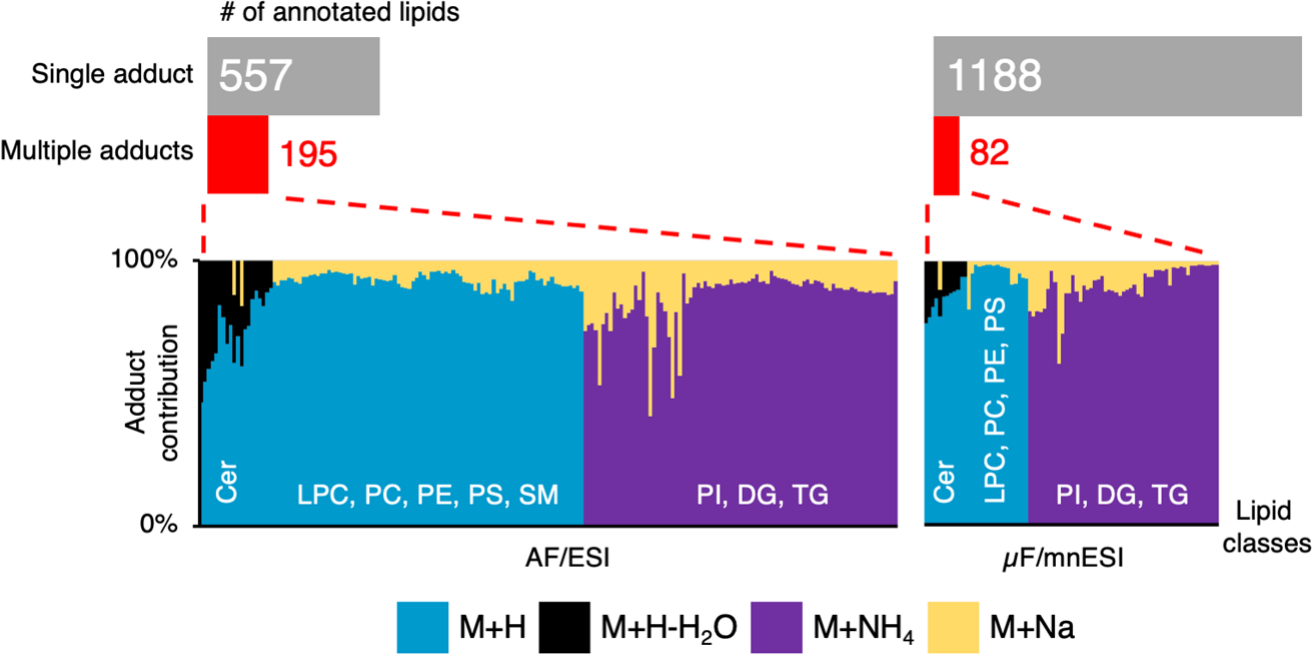
Differences in adduct formation between the established AF/ESI workflow and the μF/mnESI-MS method for untargeted lipidomics of ccRCC cells.

Considering a 15-fold reduction in injected material, an effective gain of two to four times in peak areas was achieved for compounds, such as the majority of phospholipids and triglycerides (Figure 3A–C), in line with our observations with lipid standards. A gain factor of 10- to >260-fold for specific lipid classes was observed, as demonstrated by CE 22:6, CE 22:5, and CE 18:2, unambiguously annotated with both setups (Figure S2). However, the μF/mnESI configuration displayed broad peaks with increased asymmetry (2.86 at 10% peak height compared to 1.08 for the AF setup), which negatively affected the resolution of isomers among polar lipids (Figure 3A,B). The extent of peak broadening was less pronounced for neutral lipids (1.25 vs 1.07, Figure 3C). As previously discussed, differences in adduct formation patterns were also confirmed by biological sample analysis. Only 6.5% (82) of lipids were detected as multiple ions in μF/mnESI, while the AF/ESI approach returned 25.9% (195) of such annotations (Figure 4).

### Performance of the μF/mnESI Method at the Batch Scale

**Figure 5:**
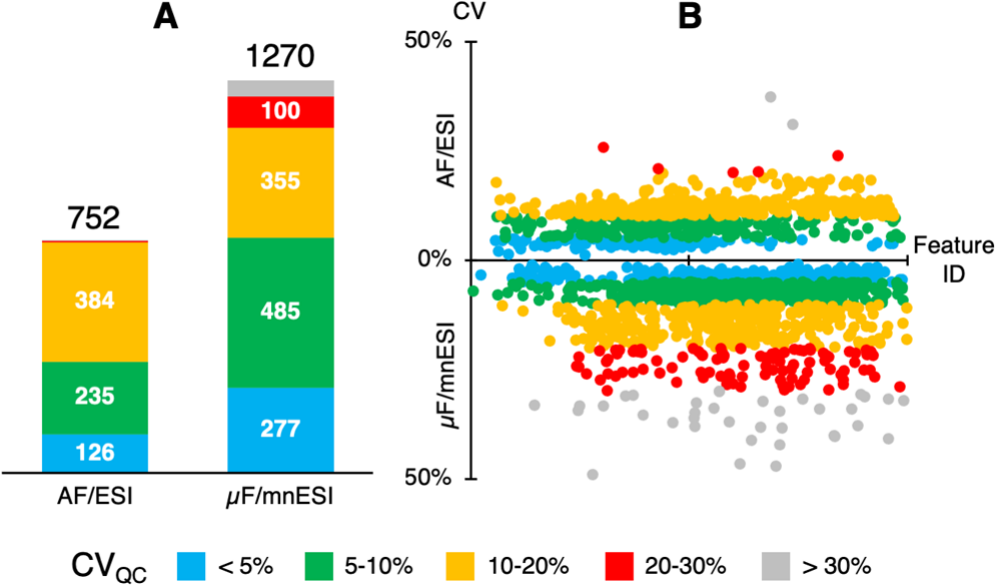
Figures of merit for a comparison of data quality between classic and μF/mnESI approaches. (A) Annotation rates. (B) Allocation of the annotated features across data sets (feature ID may be considered as an indirect measure of tR).

**Figure 6:**
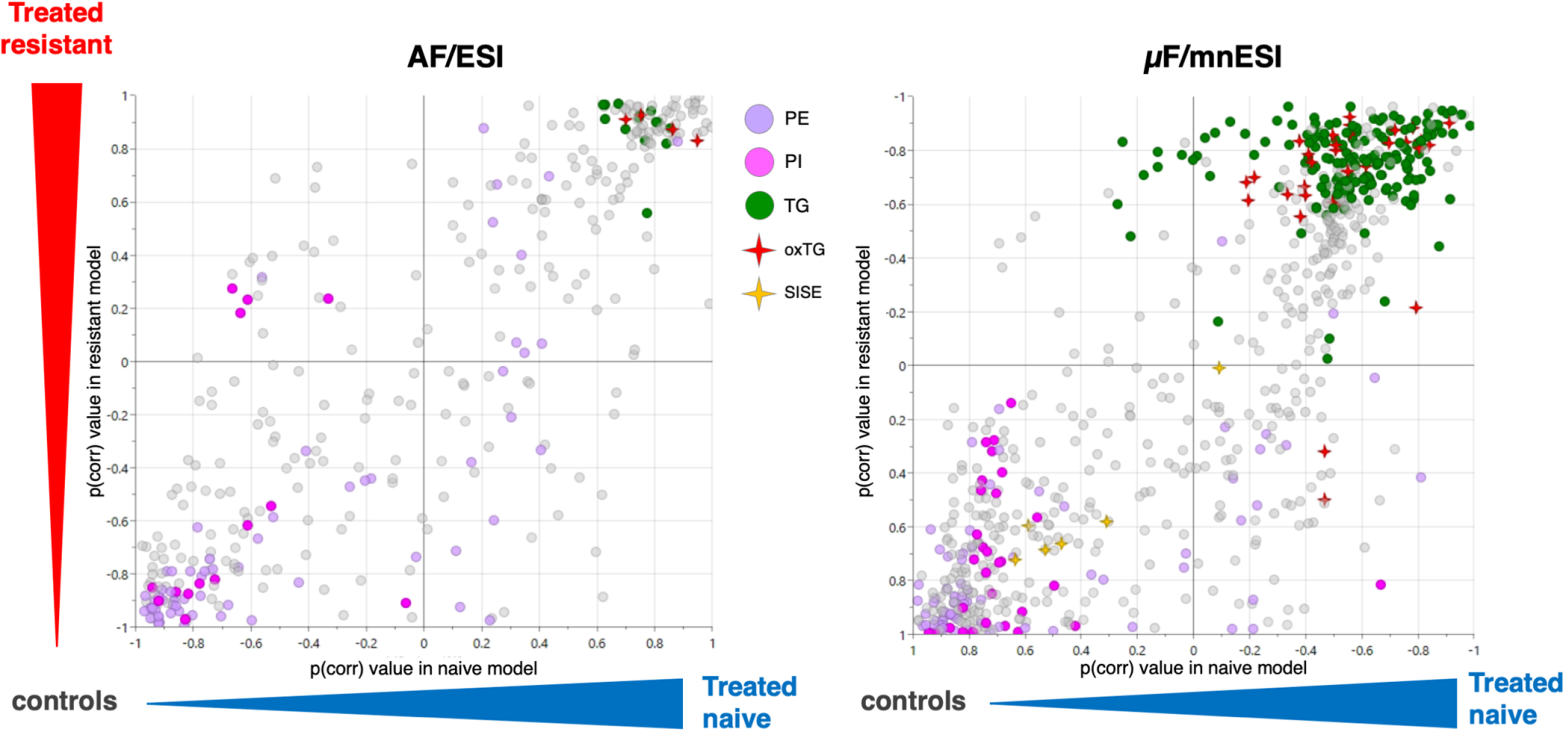
Naive vs resistant SUS plots of the AF/ESI (A) and μF/mnESI (B) data sets for the exposure to the combination therapy with lipids <10% CV. Lipids showing the strongest trends are colored by ontology, with PE as lavender dots, PI as pink dots, TGs as green dots, oxTGs as red stars, and SISE as yellow stars. OPLS-DA model of naive cells in the classic data set: (1 + 1 + 0): R2X = 0.64, Q2 = 0.88 (*n* = 6); OPLS-DA model of resistant cells in the classic data set: (1 + 1 + 0): R2X = 0.73, Q2 = 0.94 (*n* = 6); OPLS-DA model of naive cells in the μF/mnESI data set: (1 + 1 + 0): R2X = 0.60, Q2 = 0.71 (*n* = 6); OPLS-DA model of resistant cells in the μF/mnESI data set: (1 + 1 + 0): R2X = 0.59, Q2 = 0.98 (*n* = 6).

The μF/mnESI setup demonstrated stable operation across 31 h of acquisition of the 89-injection sequence. Pressure profiles were not altered across the sequence, with an average backpressure of 860 bar. Retention time shifts did not exceed ±0.3 min from the average value (Figure S3). The PCA score plot on raw data showed similar drift patterns between pooled QCs and reconditioning QCs injected after diluted QCs, suggesting minimal influence of injection-to-injection changes in the matrix (Figure S4). In terms of repeatability, the μF/mnESI method displayed excellent performance compared with conventional AF/ESI methods, as exemplified with our previous AF/ESI acquisition of the same samples. Shares of lipids with CV_QC_ < 10% were slightly higher for the μF/mnESI than for the AF/ESI setup: 60.0% (*n* = 762) vs 48.0% (*n* = 361) for μF/mnESI and AF/ESI, respectively (Figure 5A). In addition, CVs were similarly spread across the elution gradient with both setups (Figure 5B) and distributions of the measured analytical signals for both methods overlapped as bell-shaped curves with a slight shift toward higher abundances for the new setup (Figure S5). PCA score plots obtained from the conventional and the μF/mnESI data sets showed similar data structures before and after LOESS and PQN corrections (Figure S6C,D), while the miniaturized setup displayed an increased drift across the sequence prior to correction (Figure S6A,B). Outlier samples in the AF/ESI method were also observed among the most outlier samples in the μF/mnESI data set (Figure S7A). Loadings displayed the same distinct clusters of lipids in both data sets, with a bulk of phospholipids driven by the R-Tubacin-A sample, another by TGs following the N-Combination-A sample, and the last by subsets of ether TGs and DGs driven by the R-Combination-B sample (Figure S7B). SUS plots comparing the responses in treatment-naive vs treatment-resistant cells after exposure to combination therapy displayed similar overall shifts between conventional and μF/mnESI approaches (Figure 6). Most lipids did not display phenotype-specific patterns after exposure to combination therapy. Shared decreased lipids in treatment-naive and treatment-resistant cells could be highlighted in the lower left corner of the SUS plot, while common increases were located in the upper right corner in both setups. However, the μF-mnESI data set evidenced new discriminating species of interest belonging to oxidized TG and SISE classes, showing specific alterations after treatment (increased oxidized TGs and decreased SISE at the top left and bottom right of the SUS plot, respectively).

## Discussion

### μF/mnESI Provides Improved Ionization Conditions

The aim of this work was to evaluate the applicability of microflow chromatography combined with a multinozzle emitter featuring the throughput capability of a narrow-bore setup with a sensitivity comparable to that of nanospray ionization. The μF/mnESI measurements were achieved with a method run time of 19 min, shorter compared with those reported for nanoflow lipidomics methods. Retention time variations across the sequence were consistent with those in the existing literature, and the observed drift could be effortlessly corrected in the preprocessing step. Similar drifts were reported by He et al. with their nLC/NSI setup.^9^ Peak broadening constituted the main issue observed with the current setup, especially for polar lipids. We attribute this to the aqueous ligand of the stationary phase, which could interact with polar heads of the lipids. This observation was further supported by the fact that these effects were significantly less pronounced for the neutral species. To note, the Waters M-Class LC used in this work was outfitted to minimize nonspecific interactions, featuring an MP35N alloy loop and inactivated PEEKsil material for the autosampler needle and fluidic connections.

Interestingly, the μF/mnESI approach produced an enhanced ionization of around 20-fold, with striking improvements for particular lipid classes, such as sterol esters, glycosylated glycerolipids, and oxidized TGs. A decreased variation in ionization efficiency between different lipids due to the employed spraying conditions is a possible explanation for this outcome. Such a phenomenon was previously demonstrated for silicon chip devices by Hop et al. in a mixture of 25 pharmaceutical compounds of six structurally different classes. The relative responses of the NSI analysis on a chip varied only 2.2-fold, whereas the AF approach resulted in 21-fold variation across all compounds.^23^ The higher electric field strength around the nozzles generates a large excess of charge in the smaller droplets, thus improving ionization of otherwise hardly ionizable analytes.^23,24^ We also observed an increase in the detection of low-abundance isomers in cell extracts. As an example, only one recognizable chromatographic peak of TG 49:3 is detected by the AF/ESI setup (Figure 3C), whereas the μF/mnESI approach revealed additional peaks of isomers with lower abundances (Figure 3D). Given that ammonium ions are major charge carriers in ammonium acetate buffer conditions^25^ employed for the measurements, consistent detection of CEs typically ionized as NH_4_^+^ adducts^26^ was unsurprising. Höring et al. reported that CEs also increase their response both with increasing acyl chain length and number of double bonds.^27^ In accordance with their observations, only four CEs were detected by an analytical configuration, and a majority of those were with higher carbon and double bond counts (22:6, 22:5, 20:4, 18:2; all CV_QC_ = 10–15%). In turn, the μF/mnESI analysis delivered 38 CEs ranging from 14:0 to 26:5. Variation on pooled QC measurements for those species varied from 1.6 to 32.7% with 22 of those displaying CV_QC_ < 15%. Contributions from the additionally detected lipid subclasses may be rationalized in a similar fashion.

In-source fragmentation (ISF) is a well-known detrimental effect occurring mainly via collisions with the neutral gas in the RF ion guides of the mass spectrometer. The extent of the ISF is also dependent on the amount of internal energy received during the ionization process.^28,29^ It may lead to false positives in lipid annotation and identification by a wrong choice of fragments of high-abundance species as independent entities. The improvements observed in the μF/mnESI method can directly be ascribed to the alteration of the ionization mode, from pneumatically assisted ESI to NSI, as the S-lens radiofrequency level was set to the same value (70%) in both methods. This setting was reported earlier as a major factor contributing to ISF on Orbitraps in lipidomic applications.^30^ The influence of ion transfer tube temperature between both approaches (μF/mnESI = 300 °C; AF/ESI = 340 °C) may be considered a rather minor factor, due to the similar extent of ISF between TG, DG, and PE classes for both setups.

The data acquired with μF/mnESI also featured markedly fewer species with multiple adducts compared to the conventional AF/ESI configuration, observed in both the lipid standard injections and cell extracts. As the injected cell samples were desalted with the selected sample preparation procedure, the difference in the amounts of the injected matrix cannot be considered. The high number of sodiated lipids in the AF/ESI data could originate from the high amount of solvent delivered to the ion source, as it contains metal cations leeched from glass bottles.^31^ This suggestion is again supported by the reduction of sodiation upon transition from the five- to the eight-nozzle configuration, to a smaller flow rate per nozzle. In addition, faster evaporation of the smaller droplets generated by the μF/mnESI setup could create an excess of ammonium ions in the gas phase at much faster rates. Consequently, the already reduced quantity of sodium ions is further outcompeted by those of their ammonium counterparts during the ionization process.

### μF-mnESI Delivers Higher Lipidome Coverage and More Biological Information

In terms of coverage, a significantly richer lipid map featuring 1.7-fold more total annotations and 2.1-fold more lipids with CV_QC_ < 10% was produced by μF/mnESI setup from 15× less sample amount. Our observations were in line with the results reported for nLC/NSI measurements of yeast cultures^7^ and human plasma^9^ in terms of expanded lipid coverage and increased analytical sensitivity. The gain in annotation rates was, however, smaller than for nLC/NSI. It may be first attributed to decreased ion sampling at the MS inlet due to the placement of the M3 emitter further away from the transfer capillary compared to nanoflow setups. The increased voltage requirements of the silicon chip and the higher conductivity of the buffered mobile phases typical for lipidomics made the mnESI setup indeed more susceptible to discharge, as observed during method development. The difference in annotation rates may also be due to the transfer tube diameter in higher end MS models (e.g., Thermo Fusion Lumos) enabling better ion sampling.^30^ Finally, the slower collection of MS^2^ scans in our Top4-capable instrument, compared with more performant orbitraps with Top10 to Top40 options, might have played an important role. Further increase in annotation rates on our setup could however be achieved with iterative strategies.^32,33^

Finally, the μF/mnESI displayed a data structure comparable to that of the AF/ESI workflow both via unsupervised multivariate data analysis (PCA) and supervised (OPLS-DA) methods. The μF/mnESI method provided a broader coverage of lipids, with a marked increase in the coverage of sterol esters and oxidized TGs. These findings are of particular interest in the context of ccRCC pathophysiology, where higher levels of CEs and TGs were found in the cancerous renal cortex compared to healthy tissue.^34^ These lipids contributed to class separation in OPLS-DA models, as illustrated in Figure 6 with an increase in the level of oxidized TGs and a decrease in the level of SISE after exposure to combination therapy. Sitosterols are plant-derived sterols, which are currently evaluated as nutraceuticals in clinical applications.^35^ Their esterified forms have not yet been reported in mammalian *in vitro* systems. Fetal calf serum (FCS) used in cell culture preparation could be a probable source of its substrates, as it has been reported that FCS composition can greatly influence cell culture experimental outcomes.^36^ Monitoring the metabolic products of sitosterols can be of importance considering the growing clinical interest in this class of compounds. Extension of coverage to oxidized TG species with μF/mnESI is also of importance, as it indicated an otherwise hardly detectable contribution of reactive oxygen species (ROS)-dependent processes to the overall effect of cell treatment in this study. For instance, tubacin, dasatinib, and erlotinib, included in the treatment combination, are known to activate apoptotic pathways in malignant cells via increased ROS generation.^37−39^ Therefore, the presented μF/mnESI approach is indispensable for studying oxidative effects induced by drugs on lipid metabolism in cell cultures, with the aim of further characterizing the mode of action of multidrug combination therapies.

## Conclusion and Outlook

The results of this work underscore the potential of microflow-based approaches in discovery of lipidomics. In our opinion, the presented configuration provided a robust alternative to more fragile nLC systems. Not only was the initial disparity in the injected material compared to conventional high-flow methods fully addressed, but there was also a clear net positive gain in the quantifiable signal. Significant improvement in the analytical coverage was also obtained, which is of special interest to researchers working with a limited amount of biological material. Additionally, the μF/mnESI method generated a consistent data set with higher information content and quality metrics comparable to established lipidomic pipelines. Further improvements can be achieved by optimizing chromatographic conditions, particularly in terms of stationary and mobile phases as well as sample loading techniques.

## Supporting Information Available

Detailed analytical settings for both methods, the sequence layouts, summary table of the signal ratios between μF/mnESIa nd AF/ESI for all the detected deuterated standards, and data matrices for the ccRCC measurements (XLSX)
Cell culture materials, 3D ccRCC cell culture and treatment, resistance induction, and lipid extraction from 3D ccRCC cells (PDF)
