# Supplementary material for: Microflow Liquid Chromatography Coupled to Multinozzle Electrospray Ionization for Improved Lipidomics Coverage of 3D Clear Cell Renal Cell Carcinoma
Source: Anal Chem. 2025 Feb 25;97(9):5109–17. doi: 10.1021/acs.analchem.4c06337 (PMC11912133; doi:10.1021/acs.analchem.4c06337)
Supplement: Supplementary file 2 — ac4c06337_si_002.pdf [file ac4c06337_si_002.pdf]

Supporting information

Microflow liquid chromatography coupled to multinozzle electrospray ionization for improved lipidomics coverage of 3D clear cell renal cell carcinoma

Sergey Girel [1,2], Mathieu Galmiche [1,2,3], Mathis Fiault [1,2,3], Valentin Mieville [1,2], Patrycja Nowak-Sliwinska [1,2], Serge Rudaz [1,2,3*], Isabel Meister [1,2,3*]

1) School of Pharmaceutical Sciences, University of Geneva, 1211 Geneva 4, Switzerland
2) Institute of Pharmaceutical Sciences of Western Switzerland, University of Geneva, 1211 Geneva 4, Switzerland
3) Swiss Center of Applied Human Toxicology (SCAHT), 4000 Basel, Switzerland

Table of content

Supplementary materials and methods

Cell culture materials	S3
3D ccRCC cell culture and treatment	S3
Resistance induction	S3
Lipid extraction from 3D ccRCC cells	S3
AF/ESI measurements of ccRCC samples	S3

Supplementary figures

**Fig. S1**	Responses of selected deuterated standards across the dilution series for the µF/mnESI and the AF/ESI setups	S4
**Fig. S2**	Sensitivity improvement for cholesterol esters (CE) between the AF/ESI and µF/mnESI setups	S4
**Fig. S3**	Total ion current plots of 8 pooled QCs across the µF/mnESI sequence	S5
**Fig. S4**	PCA score plots of the µF/mnESI raw data and after LOESS correction and PQN normalization	S5
**Fig. S5**	Distributions of measured analytical signals	S5
**Fig S6**	PCA score plots of the AF/ESI and µF/mnESI datasets before and after LOESS and PQN corrections	S6
**Fig. S7**	PCA score plots and loadings of the AF/ESI and µFmnESI datasets, with the samples colored according to resistance	S7
**Fig. S8**	Naive *vs.* resistant SUS plots of the AF/ESI and µF/mnESI datasets for the exposure to the combination therapy	S7

CELL CULTURE MATERIALS

RPMI 1640 medium was obtained from Gibco (ThermoFisher Scientific). Fetal calf serum was purchased from Biowest (Nuaillé, France). Penicillin and streptomycin were supplied by Bioconcept (Allschwill, Switzerland). Matrigel was obtained from Corning Life Sciences (Corning, NY, USA). The drugs tacedinaline, dasatinib, erlotinib HCl and tubacin were purchased from LC Laboratories (Woburn, MA, USA).

3D ccRCC CELL CULTURE AND TREATMENT

The 786O cell line is a human cancer cell line derived of the primary ccRCC tumor of a 58-year-old male Caucasian. 786O cells have known mutations in the VHL, PTEN, TP53, and TERT genes[1]. The treatment-naive (ATCC, Manassas, VA, USA) and the sunitinib-resistant[2] 786O cells were first expanded in 2D monolayer culture in RPMI 1640 medium supplemented 10% with fetal calf serum and with 1% penicillin/streptomycin. Then, cells with addition of 2.5% Matrigel® (Corning Life Sciences, Corning, NY, USA) were seeded at a density of 1'000 cells/well in low attachment 96-well U-bottom plates (GreinerBio-One, Kremsmüster, Austria) and centrifuged to obtain homotypic spheroids. The spheroids were next cultivated for 48 hours in a humidified incubator at 37°C and 5% CO2 before performing treatment.
A total of 120 spheroids were required per each of the 3 replicates for each of the seven treatment conditions: RPMI control, DMSO control (0,18% in RPMI), tacedinaline (2.5 μM), dasatinib (0.00625 μM), erlotinib HCl (5.0 μM), tubacin (5.0 μM) and the four-drugs combination (same concentration for each drug as above). All drugs stored in working quantities at –80°C in anhydrous DMSO. On the day of treatment, drugs were thawed, diluted in RPMI and added to each well to obtain the concentration specified above. After treatment, plates were incubated for 72h before harvesting the spheroids via pipette aspiration in a 1.5 mL Eppendorf tube and left to sediment for 2 min (if sedimentation was not sufficient, a light centrifugation of 1 min at 300 g at room temperature was performed). The cell medium was removed and the spheroids were washed twice with 1 mL of 150 mM ammonium acetate. To remove the Matrigel® coating of the spheroids, the tubes were incubated on a shaker for 20 min at 4°C. The wash liquid was then removed and 1 mL of methanol: water 4:1 (v/v) was added prior to storage in liquid nitrogen.

RESISTANCE INDUCTION

As previously described, resistance was induced in the 786O cell line through a chronic exposure to 1 μM of sunitinib over the course of 32 weeks[3]. Resistance was ultimately validated by comparing the sunitinib dose-response curve of treatment-naive and chronically exposed 786O cells. CellTiter-Glo® luminescence assay (Promega, Madison, WI, USA) was used as indirect viability readout to draw the dose-response curves.

LIPID EXTRACTION FROM 3D ccRCC CELLS

To disrupt the spheroids, frozen samples stored in methanol 80% were thawed on ice, shortly vortexed and ultra-sonicated (500W, Sonics and Materials, Newtown, CT, USA) in ice bath using 10 cycles of 10 s at 30% amplitude (each cycle was interspaced with a 30 s break to avoid overheating of the bath). The samples were then stored at -20°C for 1 h to promote efficient protein precipitation before centrifugation for 15 min at 14000g/4 °C. The supernatant containing polar metabolites was transferred to a new 1.5 mL Eppendorf tube and 1 mL of cold isopropanol were added to the remaining pellet, which was resuspended by 1 min vortexing and subsequent ultra-sonication (5 cycles of 10 s at 45% amplitude in ice bath). To promote efficient lipid extraction, the tubes were then shaken on a thermomixer for 15 min at 1200 rpm/4 °C. Samples were centrifuged for 15 min at 14000g/4°C and the supernatant was collected into a new 1.5 mL Eppendorf tube and evaporated to dryness under vacuum (SpeedVac Concentrator Savant SC210A, ThermoFisher Scientific).

AF/ESI MEASUREMENTS OF THE ccRCC SAMPLES

The AF/ESI sequence used for batch-scale benchmarking was constituted of 42 biological samples and 8 pooled QCs and 1 twofold diluted QC, with a system conditioning consisting of 20 injections of QC material (sequence detailed in supplemental Table S2). This dataset was acquired without the addition of acetic acid in mobile phases, as described in supplemental Table S3, and using an exclusion list based on blank samples.

---

[1] Bairoch, A. The Cellosaurus, a Cell-Line Knowledge Resource. *J Biomol Tech.* **2018** 29(2):25-38. https://doi.org/10.7171/jbt.18-2902-002

[2] Rausch, M.; Weiss, A.; Achkhanian, J.; Rotari, A.; Nowak-Sliwinska, P. Identification of low-dose multidrug combinations for sunitinib-naive and pre-treated renal cell carcinoma. *Br J Cancer* **2020** 123, 556–567. https://doi.org/10.1038/s41416-020-0890-y

[3] Weiss, A.; Le Roux-Bourdieu, M.; Zoetemelk, M.; Ramzy, G.M.; Rausch, M.; Harry, D.; Miljkovic-Licina, M.; Falamaki, K.; Wehrle-Haller, B.; Meraldi, P.; Nowak-Sliwinska, P. Identification of a Synergistic Multi-Drug Combination Active in Cancer Cells via the Prevention of Spindle Pole Clustering. *Cancers* **2019** 11(10):1612. https://doi.org/10.3390/cancers11101612

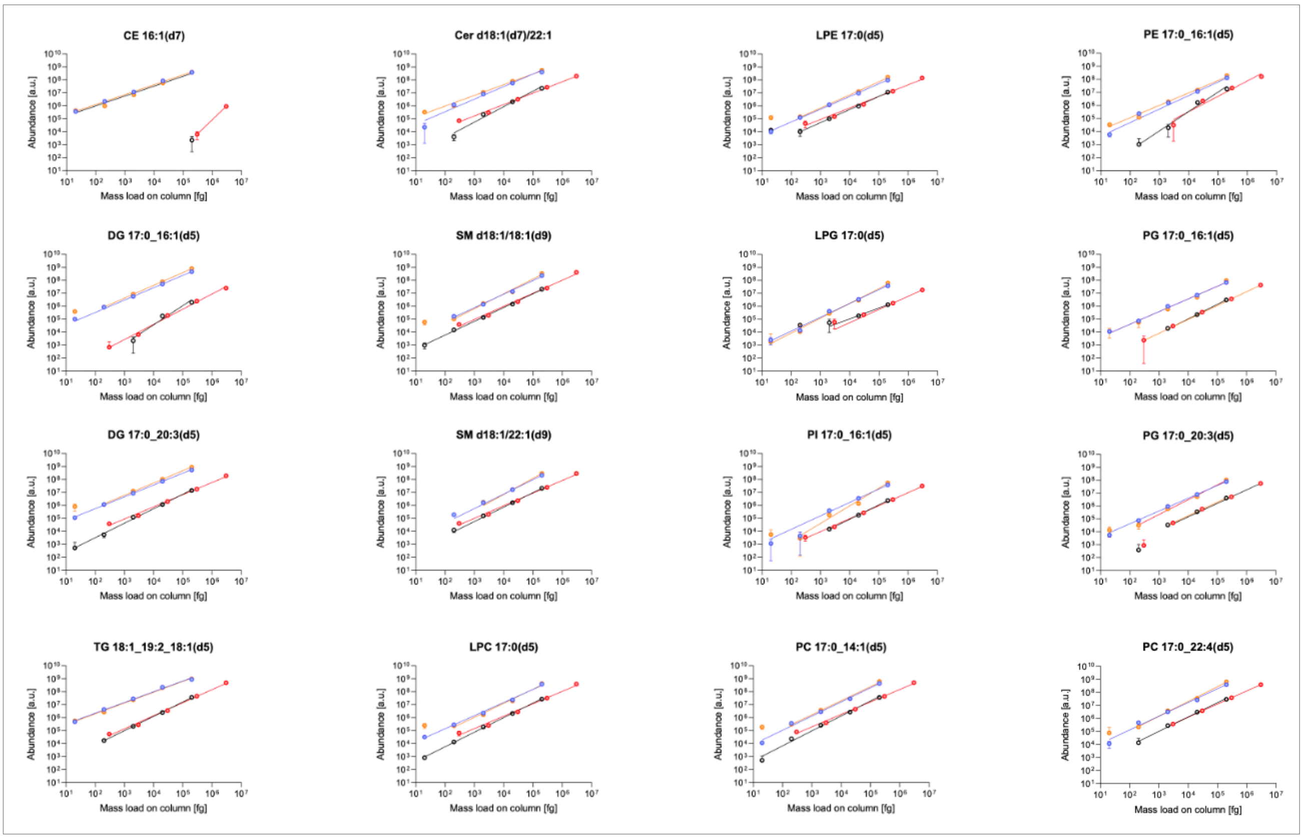

Fig. S1 Responses (peak areas) of selected deuterated standards across the dilution series for each of the configurations tested (µF/mnESI with 5- or 8-nozzle emitter and AF/ESI; plotted with GraphPad Prism v.9.5.1; mean, SD, log10-scale).

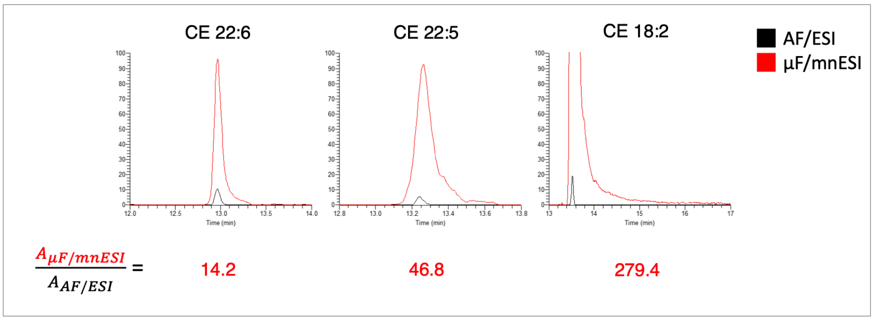

Fig. S2 Sensitivity improvement for cholesterol esters (CE) between AF/ESI approach (black trace) and µF/mnESI setup (red trace). All species were detected as [M+NH4]+ ions. Selected ion chromatograms extracted within 3 ppm range.
Chromatogram alignment made by an offset applied to the trace of the AF/ESI method.

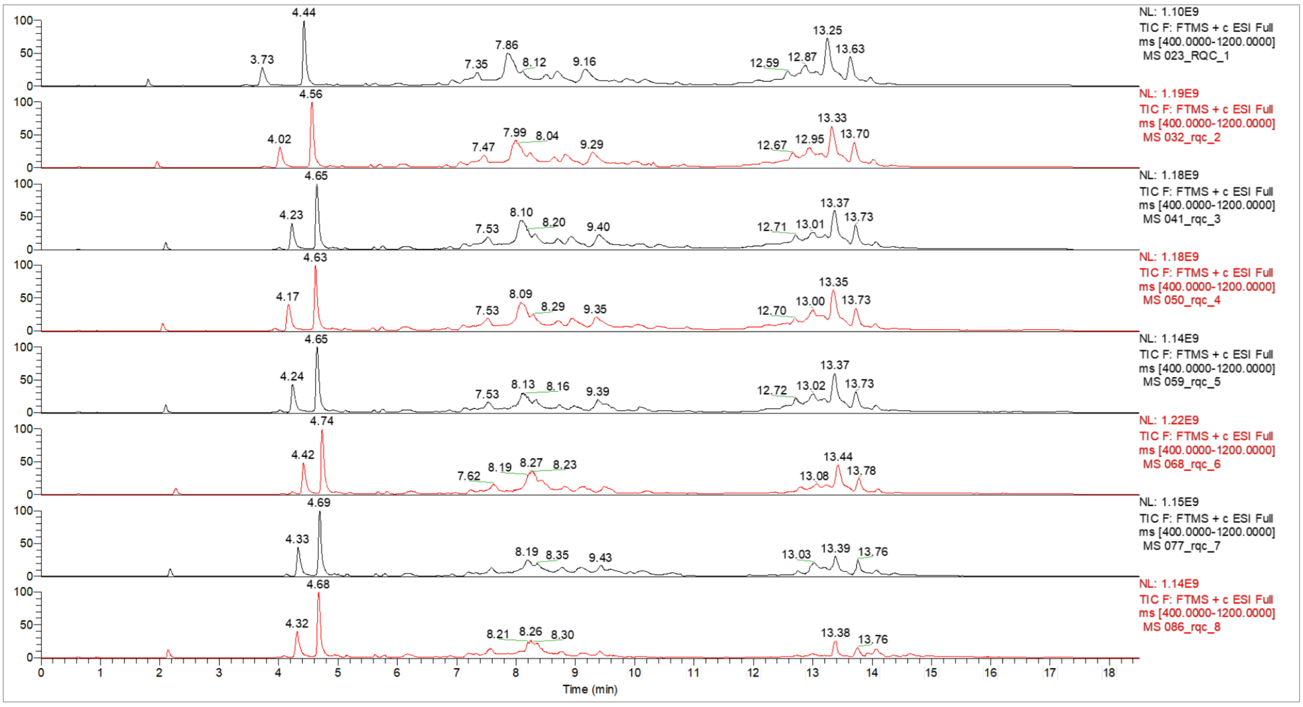

Fig. S3 Total ion current plots of 8 reconditioning pooled QCs injected after each 2-fold diluted QCs across the sequence demonstrating the minor systematic retention time shift observed in the analysis performed on the µF/mnESI setup (± 0.3 min).

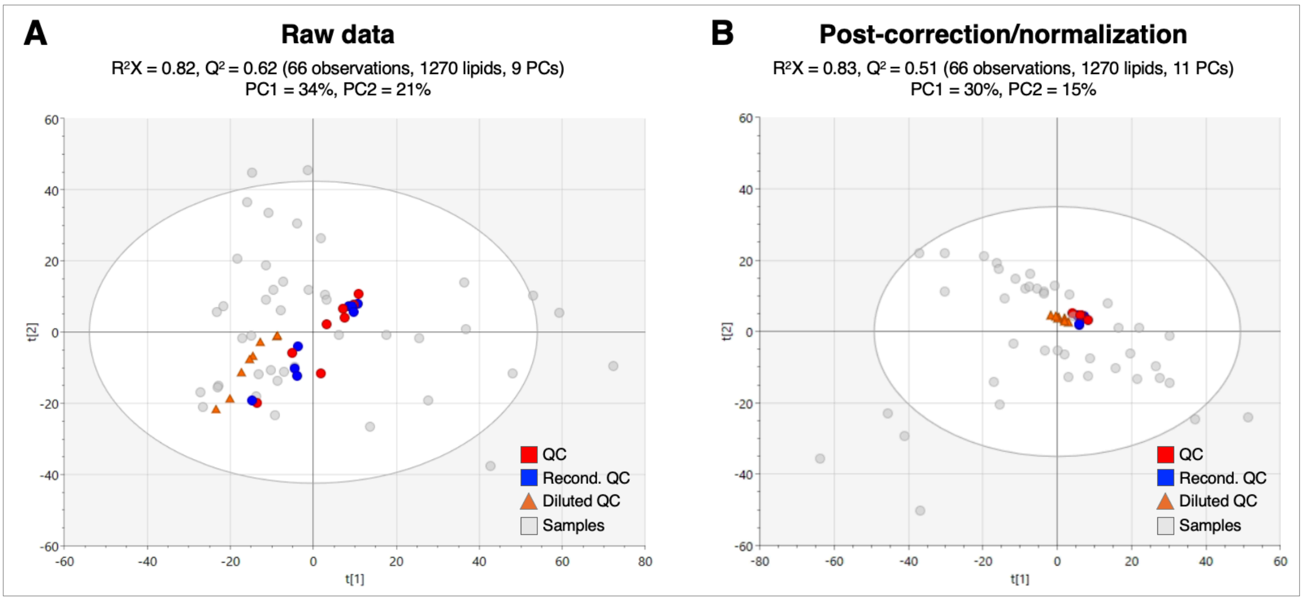

Fig. S4 PCA score plots of the µF/mnESI raw data (A) and after LOESS correction and PQN normalization (B) highlighting the clustering of reconditioning QC samples (blue dots) injected after the diluted QCs (in orange triangles) compared to QCs injected prior diluted QCs (red dots).

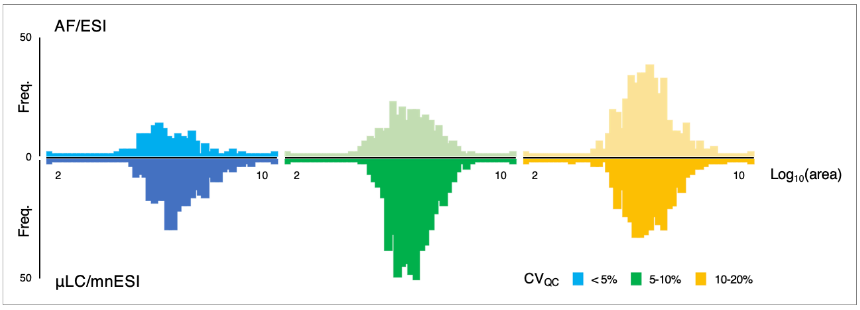

Fig. S5 Distribution of measured analytical signals (normalized to 50 bins) for AF/ESI and µF/mnESI separated by CV ranges.

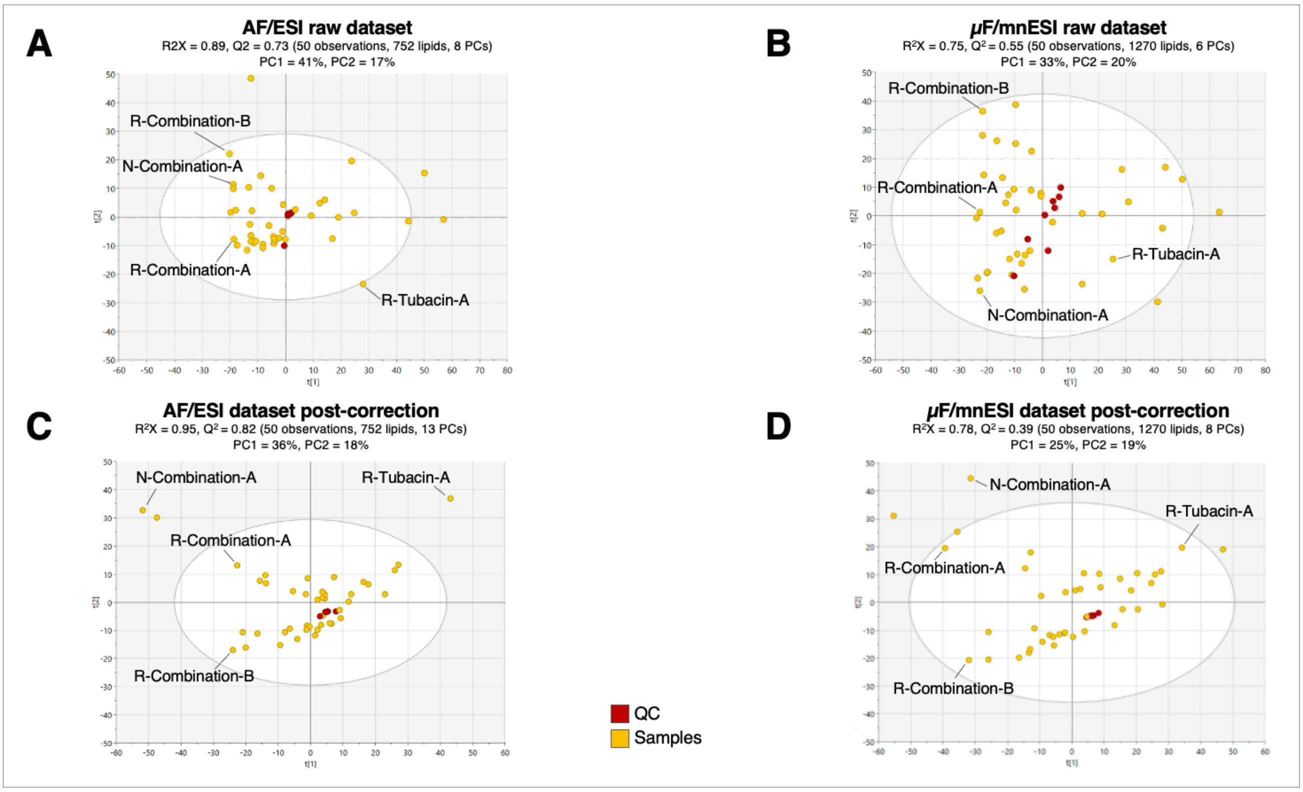

Fig S6 PCA score plots of the AF/ESI and µF/mnESI datasets before (A, B) and after LOESS and PQN corrections (C, D), with the QC samples colored in red and study samples in yellow.

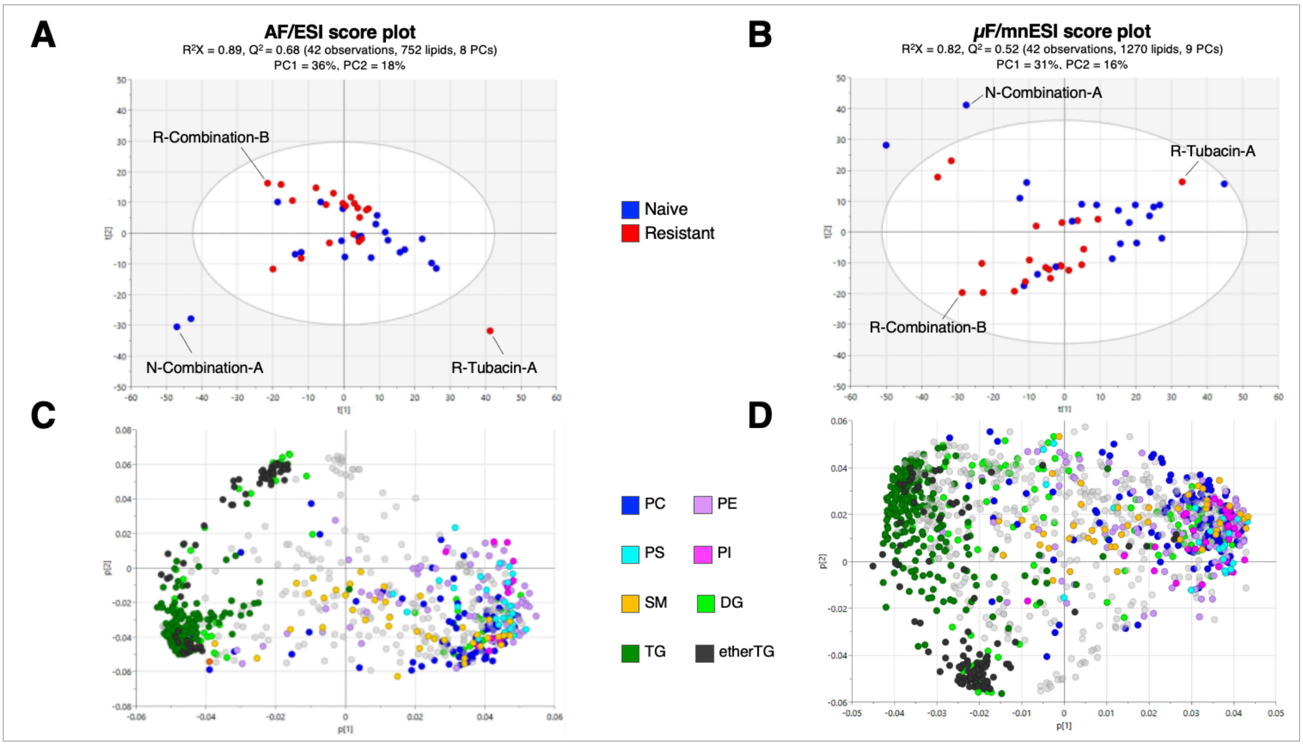

Fig. S7 PCA score plots of the AF/ESI (A) and µFmnESI (B) datasets, with the samples colored according to resistance (blue: naive cells, red: sunitinib-resistant cells. Corresponding loading plots of the AF/ESI (C) and µFmnESI (D) datasets with lipids colored according to ontology.

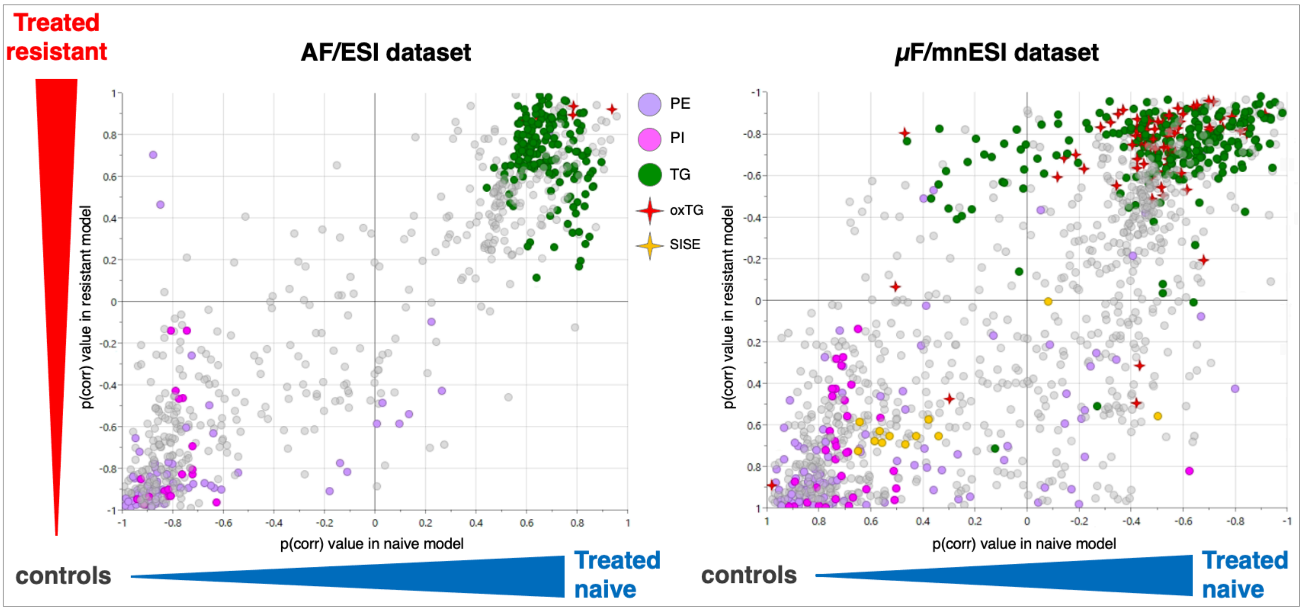

Fig. S8 Naive vs resistant SUS plots of the AF/ESI (A) and µF/mnESI (B) datasets for the exposure to the combination therapy. Lipids showing the strongest trends are colored by ontology, with PE as lavender dots, PI as pink dots, TGs as green dots, oxTGs as red stars and SISE as yellow stars. OPLS-DA model of naive cells in the classic dataset: (1+1+0): R2X = 0.70, Q2 = 0.84 (n=6); OPLS-DA model of resistant cells in the classic dataset: (1+1+0): R2X = 0.83, Q2 = 0.96 (n=6); OPLS-DA model of naive cells in the µLC-mnESI dataset: (1+1+0): R2X = 0.60, Q2 = 0.75 (n=6); OPLS-DA model of resistant cells in the µLC-mnESI dataset: (1+1+0): R2X = 0.62, Q2 = 0.98 (n=6)
